# AAV-Delivered *Tulp1* Supplementation Therapy Targeting Photoreceptors Provides Minimal Benefit in *Tulp1−/−* Retinas

**DOI:** 10.3389/fnins.2020.00891

**Published:** 2020-08-27

**Authors:** Arpad Palfi, Adlet Yesmambetov, Sophia Millington-Ward, Ciara Shortall, Pete Humphries, Paul F. Kenna, Naomi Chadderton, G. Jane Farrar

**Affiliations:** Department of Genetics, School of Genetics and Microbiology, Trinity College Dublin, Dublin, Ireland

**Keywords:** retina, eye, mouse, disease model, inherited retinal degeneration, *Tulp1*, retinitis pigmentosa, AAV

## Abstract

With marketing approval of the first ocular gene therapy, and other gene therapies in clinical trial, treatments for inherited retinal degenerations (IRDs) have become a reality. Biallelic mutations in the tubby like protein 1 gene (*TULP1*) are causative of IRDs in humans; a mouse knock-out model (*Tulp1−/−*) is characterized by a similar disease phenotype. We developed a *Tulp1* supplementation therapy for *Tulp1−/−* mice. Utilizing subretinal AAV2/5 delivery at postnatal day (p)2–3 and rhodopsin-kinase promoter (*GRK1P*) we targeted *Tulp1* to photoreceptor cells exploring three doses, 2.2E9, 3.7E8, and 1.2E8 vgs. *Tulp1* mRNA and TULP1 protein were assessed by RT-qPCR, western blot and immunocytochemistry, and visual function by electroretinography. Our results indicate that TULP1 was expressed in photoreceptors; achieved levels of *Tulp1* mRNA and protein were similar to wild type levels at p20. However, the thickness of the outer nuclear layer (ONL) did not improve in treated *Tulp1−/−* mice. There was a small and transient electroretinography benefit in the treated retinas at 4 weeks of age (not observed by 6 weeks) when using 3.7E8 vg dose. Dark-adapted mixed rod and cone a- and b-wave amplitudes were 24.3 ± 13.5 μV and 52.2 ± 31.7 μV in treated *Tulp1−/−* mice, which were significantly different (*p* < 0.001, *t*-test), from those detected in untreated eyes (7.1 ± 7.0 μV and 9.4 ± 15.1 μV, respectively). Our results indicate that *Tulp1* supplementation in photoreceptors may not be sufficient to provide robust benefit in *Tulp1−/−* mice. As such, further studies are required to fine tune the *Tulp1* supplementation therapy, which, in principle, should rescue the *Tulp1−/−* phenotype.

## Introduction

Biallelic mutations in tubby like protein 1 gene (*TULP1*) are responsible for rare forms of early onset, severe, autosomal recessive retinal degenerations, typically registered as retinitis pigmentosa 14 (RP14) or Leber congenital amaurosis 15 (LCA15) (Hagstrom et al., 1998; Mataftsi et al., 2007; Jacobson et al., 2014; RetNet - Retinal Information Network, 2019). *TULP1* is one of more than 300 genes known to cause inherited retinal degenerations (IRDs) (Duncan et al., 2018; RetNet - Retinal Information Network, 2019), which cumulatively affect 1 in 3000 individuals (Sahel et al., 2015; Whelan et al., 2020).

In mice, *Tulp1* is expressed at high levels in photoreceptors and is primarily distributed to the inner segments and synaptic terminals (Hagstrom et al., 1998; Ikeda et al., 2000; Xi et al., 2005), where it is essential to photoreceptor functions, such as protein transport between the inner and outer segments (Grossman et al., 2011) and synaptic transmission (Grossman et al., 2009). Originally, it was believed that *Tulp1* was specific to photoreceptors (Hagstrom et al., 1998; Ikeda et al., 2000), however, there is emerging evidence that it is also expressed, albeit at lower levels, in other retinal cell types, such as progenitor, ganglion (Milam et al., 2000), and RPE cells (Caberoy et al., 2010; Whitmore et al., 2014; Palfi et al., 2020).

Tubby like protein 1 gene-linked retinal degenerations, along with other forms of IRDs, are disorders with unmet therapeutic interventions (Sahel et al., 2015; Farrar et al., 2017) and are responsible for significant personal, social, and economic burdens (Smith et al., 2015; Retina International, 2019). With FDA/EMA approval of Luxturna^TM^, the first ocular gene therapy in 2017/18, interest in development of gene therapies for IRDs is substantial. In preclinical proof of principle studies, animal models of IRDs (Ziccardi et al., 2019; Collin et al., 2020) have been extremely useful and ultimately contributed to paving the way toward clinical trials for many retinal gene therapies (ClinicalTrials.gov); reviewed by Dalkara et al. (2016) and Trapani and Auricchio (2019). Many preclinical studies have highlighted the value of adeno associated virus (AAV) as a means of gene delivery and have demonstrated benefit at molecular, histological and functional levels of AAV-delivered gene therapies (among others, Millington-Ward et al., 2011; Chadderton et al., 2013; Schön et al., 2017; Cideciyan et al., 2018). Such studies prompted us to explore of a similar therapeutic approach for *TULP1*-linked retinal degenerations.

A mouse model of *TULP1*-linked IRDs (*Tulp1−/−* mouse) has been generated with a targeted disruption of *Tulp1* (Hagstrom et al., 1999; Ikeda et al., 2000). The course of retinal degeneration in *Tulp1−/−* mice is similar to that found in *TULP1*-linked human IRD patients. Symptoms in *Tulp1−/−* mice present early in life and include shortening of photoreceptor segments (Ikeda et al., 2000), abnormal photoreceptor synaptic architecture (Grossman et al., 2009) and progressive photoreceptor apoptosis (Ikeda et al., 2000). The function of *Tulp1−/−* retinas, as assessed by electroretinography (ERG) is also significantly compromised from early on (Hagstrom et al., 1999; Ikeda et al., 2000; Grossman et al., 2009).

As *Tulp1* is predominantly expressed in photoreceptor cells, in this study, we aimed to assess the benefit of photoreceptor-targeted *Tulp1* supplementation in *Tulp1−/−* mice, which mimics a potential therapeutic approach for *TULP1*-linked patients. A strategy akin to those we applied successfully to *RHO* replacement in mouse models (O’Reilly et al., 2007; Palfi et al., 2010; Millington-Ward et al., 2011) was devised; similar strategies have been beneficial in other *RHO* models (Lewin et al., 2014; Cideciyan et al., 2018). As the *Tulp1−/−* retina deteriorates rapidly, AAV-delivered *Tulp1* supplementation was administered subretinally to *Tulp1−/−* mice at postnatal day (p)2–3 and effects analyzed from p20. In spite of efficient expression of *Tulp1* mRNA and TULP1 protein in photoreceptors, the therapeutic strategy provided only marginal and transient, though statistically significant, functional benefit in *Tulp1−/−* mice.

## Materials and Methods

### Constructs and AAV Production

**FIGURE 1:**
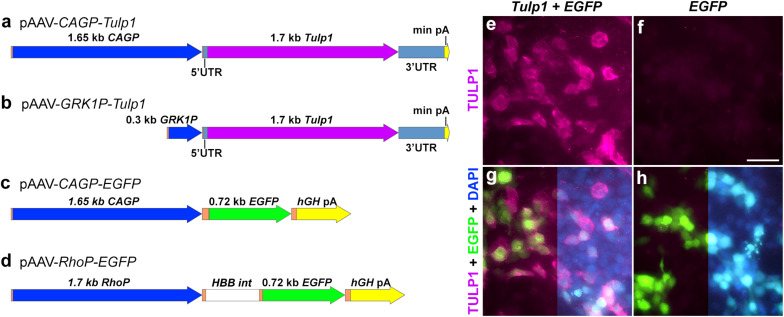
Plasmid constructs and *in vitro* TULP1 expression analysis. **(a–d)** pAAV plasmid constructs. *CAGP*, *CAG* promoter; min pA, minimal poly(A); *GRK1P*, rhodopsin kinase promoter; *RhoP*, rhodopsin promoter; *hGH* pA, human growth hormone poly(A); *HBB int*, b-globin intron; orange label, vector sequences. **(e–h)** HEK293 cells were transiently transfected with 1:1 ratio of pAAV-*CAGP-Tulp1* (*Tulp1*) and pAAV-*CAGP-EGFP* (*EGFP*) or pAAV-*CAGP-EGFP* (*EGFP*) alone. TULP1 was detected by immunocytochemistry (purple), EGFP by native fluorescence (green) and nuclei were counterstained with DAPI (blue; right sides of **g** and **h**). Scale bar **(f)**: 40 mm.

The pAAV-*CAGP-Tulp1* vector comprised a *CAG* promoter (Jun-ichi et al., 1989) (*CAGP*); 1650 bp; *CMVIE* enhancer, 661–1024 bp from X03922; chicken-*ACTB* proximal promoter/intron 1 (251–1542 bp of X00182; 1538–1539 bp AG was changed to CA), codon optimized *Tulp1* cDNA + UTRs (NM_021478; 1632 bp) and a minimal rabbit β-globin poly(A) [49 bp; (Levitt et al., 1989)]. These were assembled in pcDNA3.1+ plasmid vector (Thermo Fischer Scientific), and subsequently cloned into pAAV-MCS (Agilent Technologies). To generate pAAV-*CAGP-EGFP* vector, the *EGFP* coding sequence (U55761) and *hGH Poly(A)* (from pAAV-MCS) was substituted for *Tulp1* above. pAAV-*GRK1P-Tulp1* was created by replacing the *CAGP* in pAAV-*CAGP-Tulp1* with *GRK1P* (1793–2087 bp of AY327580.1, (Beltran et al., 2010). pAAV-*RhoP-EGFP* was cloned as described and utilized a 1.7 kb *Rho* promoter (Palfi et al., 2010). The final constructs; pAAV-*CAGP-Tulp1*, pAAV-*GRK1P-Tulp1*, pAAV-*CAGP-EGFP*, and pAAV-*RhoP-EGFP* were sequence verified and are depicted in Figure 1. Recombinant AAV2/5 (Hildinger et al., 2001) viruses were generated using a triple transfection method (Xiao et al., 1998), then purified by differential precipitation and cesium gradient centrifugation (Bennicelli et al., 2008; Ayuso et al., 2010; Palfi et al., 2010). Genomic titers (viral genomes/ml; vg/ml) were determined by qPCR (Rohr et al., 2005).

### Animals and AAV Delivery

*Tulp1−/−* mice on C57BL/6J background (B6.129 × 1-Tulp1tm1Pjn/Pjn; The Jackson Laboratory) (Hagstrom et al., 1999; Ikeda et al., 2000) and wild type C57BL/6J (wt) mice were used in this study. Mice were maintained under specific pathogen free (SPF) housing conditions and both sexes were used for experiments. Animal welfare complied with the Directive 2010/63/EU; Protection of Animals Used for Scientific Purposes, Regulations 2012 [S.I. No. 543 of 2012] and the Association for Research in Vision and Ophthalmology (ARVO) Statement for the Use of Animals in Ophthalmic and Vision Research. AAV was subretinally delivered in 0.6 μl PBS (supplemented with 0.001% Pluronic F68)/eye to pups at p2–3 as previously described (Palfi et al., 2010).

### Transfection, RT-qPCR, Western Blot and Electroretinography

Human embryonic kidney cells (HEK293; accession number CRL-1573; ATCC) were transfected using Lipofectamine 3000 (Thermo Fischer Scientific), according to the manufacturer’s instructions. Briefly, 5 × 10^5^ cells per well were seeded onto 6-well plates containing 1 ml Dulbecco’s modified Eagle medium (DMEM) supplemented with 10% calf serum, 2 mM glutamine and 1 mM sodium pyruvate and incubated overnight at 37°C. Cells were washed twice with PBS, then each well was transfected with 0.5 mg plasmid DNA in Opti-MEM (Thermo Fischer Scientific) and incubated for 4 h at 37°C, then media was replaced with complete DMEM. Cells were analyzed 48 h post-transfection. RT-qPCR was carried out as described (O’Reilly et al., 2007). Protein extraction and western blot was performed according to Grossman et al. (2014). Electroretinography (ERG) analysis was completed as described in O’Reilly et al. (2007) except gold wire electrodes were used instead of contact lens electrodes.

### Immunohistochemistry, Microscopy, Morphometry and Statistical Analysis

Mice were sacrificed, eyes enucleated and fixed in 4% paraformaldehyde in PBS at 4°C overnight. Eyes were washed in PBS, cryoprotected in 10%, 20%, and 30% sucrose in PBS, embedded in OCT (VWR), cryosectioned (12 μm), thaw-mounted onto polysine slides (Thermo Scientific) and stored at −20°C. For immunocytochemistry, sections adjacent to the optic nerve head (±200 μm) were blocked in 5% donkey serum, 0.3% Triton-X-100 in PBS (blocking solution) for 2 h at room temperature, then incubated with primary TULP1 antibody (M-tulp1N; Stephanie A. Hagstrom, Cleveland Clinic, Cleveland, OH, United States) in 1:200 dilution in blocking solution at 4°C overnight, then with secondary anti-rabbit antibody conjugated with Cy3 (Jackson ImmunoResearch Laboratories) in 1:400 dilution in blocking solution at room temperature for 2 h. Washes after the primary and secondary antibody incubations were carried out in PBS (3 × 10 min). Finally, nuclei were counterstained with DAPI followed by 3 × 5 min washes in PBS. Fluorescent microscopy was carried out utilizing an Olympus IX83 inverted motorized microscope (cellSens v1.9 software) equipped with a SpectraX LED light source (Lumencor) and an Orca-Flash4.0 LT PLUS/sCMOS camera (Hamamatsu). Multi-channel gray-scale images were assigned with fluorescence colors and channels superimposed. 10× magnification pan-retinal images were produced from images with lateral frames stitched together in cellSens. Measurements of outer nuclear layer (ONL) thickness were taken in the stitched images from the central half of the retinas; four sections/eye were analyzed, and four measurements per section were made using cellSens software (Olympus). Representative images for figures were exported from cellSens as individual fluorescence channels and post-processed in Photoshop (Adobe). In a given observation method, the same settings/operations were applied to all images both in cellSens and Photoshop. One-way analysis of variance (ANOVA) with a Tukey’s multiple comparison *post hoc* test and unpaired *t*-test were used for statistical analyses (Prism 8, GraphPad); *p*-values of less than 0.05 were considered statistically significant.

## Results

Tubby like protein 1 gene and *EGFP* expression vectors were constructed using ubiquitous and photoreceptor specific promoters and the mouse *Tulp1* cDNA (Figures 1a–d and as detailed in “Materials and Methods”). pAAV-*CAGP-Tulp1* and pAAV-*CAGP-EGFP* (control) were either transfected into HEK293 cells at a 1:1 ratio or the control vector alone and expression of TULP1 in pAAV-*CAGP-Tulp1*-tranfected cells confirmed using TULP1 immunocytochemistry; expression of EGFP was detected using native fluorescence (Figures 1e–h).

AAV-*GRK1P-Tulp1* (+ tracer AAV-*RhoP-EGFP*) was then administered subretinally to *Tulp1−/−* pups at three doses at p2–3; 2.2E9 vg AAV-*GRK1P-Tulp1* + 2.7E7 vg AAV-*RhoP-EGFP* (high dose, HD), 3.7E8 vg AAV-*GRK1P-Tulp1* + 2.7E7 vg AAV-*RhoP-EGFP* (middle dose, MD), and 1.2E8 vg AAV-*GRK1P-Tulp1* + 1.8E7 vg AAV-*RhoP-EGFP* (low dose, LD). Control eyes received 2.7E7 vg AAV-*RhoP-EGFP*. C57BL/6J wild type (wt) mice were used as healthy controls. AAV-*RhoP-EGFP* was omitted in injections for ERG analysis. We found that the p2–3 timepoint resulted in the highest number of transduced photoreceptor cells when delivering AAV2/5 subretinally in *Tulp1−/−* mice. While there are more photoreceptor cells available at p5–7, by this time the progressive degeneration of photoreceptor segments makes the subretinal injection physically challenging and less efficient in *Tulp1−*/*−* mice. Retinas were analyzed by RT-qPCR, western blot, histology and ERG at various timepoints post-delivery.

**FIGURE 2:**
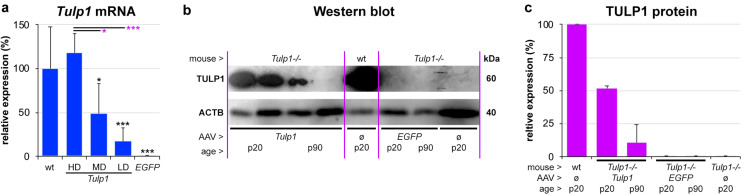
*In vivo* expression analysis of *Tulp1* and TULP1. *Tulp1–/–* mice were subretinally injected with LD, MD and HD of AAV-*GRK1P-Tulp1* (LD, MD, HD) or AAV-*RhoP-EGFP* (*EGFP*) at p2–3. **(a)** *Tulp1* mRNA expression (*Actb* used as internal control) was analyzed using RT-qPCR in RNA samples from AAV-*GRK1P-Tulp1* (*Tulp1*) transduced *Tulp1–/–* retinas at p20, bars represent mean + SD (*n* = 4–7). Wild type (wt) and AAV-*RhoP-EGFP* (*EGFP*) transduced retinas were used as controls. ^∗^*p* < 0.05, ^∗∗∗^*p* < 0.001; black asterisks indicate differences compared to wt while purple asterisks indicated differences compared to HD (ANOVA). **(b)** TULP1 protein expression was detected following subretinal delivery of MD of AAV-*GRK1P-Tulp1* (*Tulp1*) using western blot analysis at p20 and p90. Uninjected (ø) wt and *Tulp1–/–* retinas, and *Tulp1–/–* retinas injected with AAV-*RhoP-EGFP* (*EGFP*) were used as controls. Three retinas were combined/lane, 30 mg protein/lane was analyzed and ACTB was used as a loading control. **(c)** Blots were quantified utilizing ImageJ; bars represent mean + SD (*n* = 1–2).

Tubby like protein 1 gene mRNA expression was assessed using RT-qPCR at p20 (*n* = 4–7) and *Actb* mRNA was used as an internal control (Figure 2a). Relative expression levels from the AAV dose curve were 16.9 ± 15.5% (LD), 48.6 ± 34.7% (MD), and 117.7 ± 22.1% (HD), the latter being similar to the wt *Tulp1* mRNA expression level (100.0 ± 47.7%). Expression levels between HD/MD and HD/LD were significantly different, *p* < 0.05 and *p* < 0.001, respectively. However, given a typical 40–50% pan-retinal coverage after subretinal injection with AAV2/5 at p2–3, the MD of AAV-*GRK1P-Tulp1* provided similar mRNA expression to wt levels of *Tulp1* mRNA (Figure 2a). There was no *Tulp1* mRNA detected in the control, *EGFP* transduced *Tulp1−/−* retinas (0.1 ± 0.07%).

Tubby like protein 1 protein expression was evaluated by western blot analysis in retinas transduced with the MD dose of AAV-*GRK1P-Tulp1* at p20 and p90 (Figures 2b,c) and wt, AAV-*RhoP-EGFP* transduced and untransduced *Tulp1−/−* retinas were used as controls. Six *Tulp1−/−* retinas were transduced with AAV-*GRK1P-Tulp1* for both p20 and p90 timepoints. As few transduced retinas were positive at p90, three retinas were combined; to use the same methodology, the other samples were also prepared by combining three retinas; 30 mg protein/lane was run. Expression of TULP1 in AAV-*hRKP-Tulp1* transduced retinas was confirmed by western blot analysis and the blots were quantified; ACTB label was used as loading control. The MD of AAV-*hRKP-Tulp1* provided 51.9 ± 1.5% of wt TULP1 protein level at p20, however, this was substantially reduced by p90 (10.6 ± 13.6%; Figures 2b,c). There was no TULP1 protein in the AAV-*RhoP-EGFP* transduced (<0.1%) or untransduced *Tulp1−/−* retinas (0.16%; Figure 2b).

**FIGURE 3:**
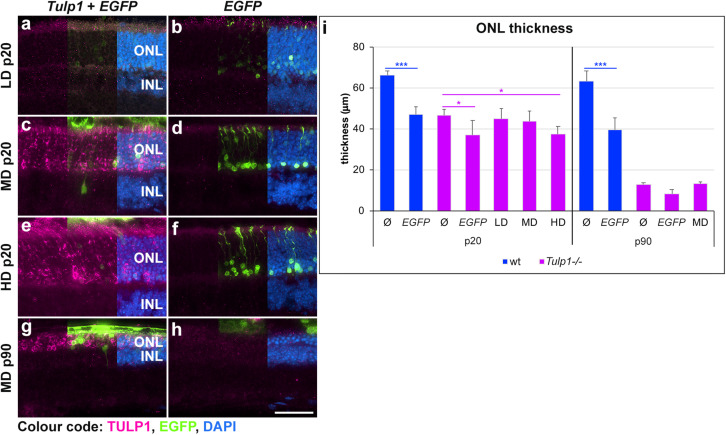
Histological evaluation of *Tulp1* supplementation in *Tulp1–/–* retinas. *Tulp1–/–* pups were injected subretinally with LD, MD and HD doses of AAV-*GRK1P-Tulp1* + AAV-*RhoP-EGFP* as a marker (*Tulp1* + *EGFP*) or AAV-*RhoP-EGFP* (*EGFP*) as injection controls and their retinas analyzed at p20 and p90 (*n* = 4–6). Age-matched, uninjected (ø) wt and *Tulp1–/–* retinas were used as uninjected controls. **(a–h)** Eyes were cryosectioned and TULP1 immunocytochemistry (purple) carried out; EGFP (green) was detected by native fluorescence and nuclei were counterstained with DAPI (blue). Scale bar **(h)**: 40 mm. The thickness of the ONL in the transduced areas of the central retina was measured using cellSens (Olympus) and is shown in the bar chart; bars represent mean + SD **(i)**. ONL, outer nuclear layer; INL, inner nuclear layer. *p < 0.05, ***p < 0.001 blue asterisks indicate differences between ø and treated wt retinas, while purple asterisks indicated differences between uninjected (ø) and treated *Tulp1–/–* retinas (ANOVA).

Following AAV-*GRK1P-Tulp1* delivery at p2–3, effects of *Tulp1* supplementation were evaluated by histological analysis (Figure 3 and Supplementary Figure S1). No obvious signs of major toxicity relating to the AAV delivery were observed in the treated *Tulp1−/−* retinas (Figure 3). Tulp1 immunohistochemistry indicated TULP1 expression in the ONL of transduced areas of retinas (Figures 3a,c,e,g). Expression of TULP1 was analyzed in the LD, MD, and HD groups at p20 (expression increased with dose) and with the MD at p90. By p90 the ONL contained only 0–3 rows of cells (Figure 3 and Supplementary Figure S1) and in some retinas the TULP1 labeled area was minimal or not present at all; in this case, the transduced area was identified by EGFP (marker for transduction) expression in the adjacent retinal pigment epithelium (RPE). The thickness of the ONL was measured in the transduced areas of the central retina (Supplementary Figure S1); quantification is given in a bar chart (Figure 3i). No benefit by *Tulp1* supplementation was observed in ONL thickness or the structure of the photoreceptor layer (Figure 3 and Supplementary Figure S1). Note that delivery of pAAV-*RhoP-EGFP* had a small negative effect on the ONL thickness compared to uninjected eyes both in wt and *Tulp1−/−* retinas (Figure 3 and Supplementary Figure S1).

**FIGURE 4:**
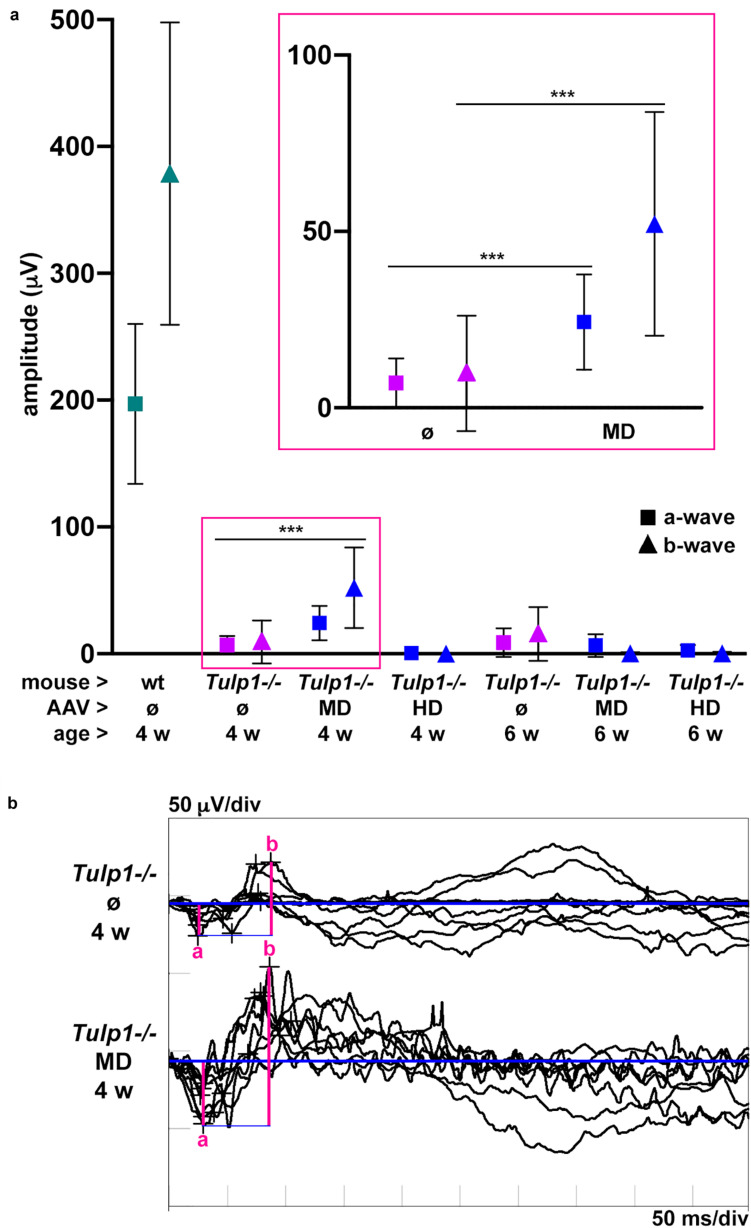
Analysis of dark-adapted, mixed rod and cone ERGs of *Tulp1* supplementation in *Tulp1–/–* retinas. *Tulp1–/–* mice were subretinally injected with MD or HD of AAV-*GRK1P-Tulp1* at p2–3; uninjected (ø) wt and Tulp1–/– retinas were used as controls (*n* = 4–16); ERG analysis was performed at 4 and 6 weeks of age. **(a)** Mean amplitudes of dark-adapted, mixed rod and cone responses to the maximal flash intensity (0 dB); a- (■) and b- (▲) waves are presented for wt (green), untreated (magenta) and treated (blue) Tulp1–/– mice in a dot plot; symbols represent mean ± SD. Data from untreated and MD-treated eyes at 4 weeks are also given magnified in the insert. ***p < 0.001 (*t*-test). **(b)** Traces of dark-adapted, mixed rod and cone responses to the maximal flash intensity (0 dB) for MD-treated and untreated *Tulp1–/–* retinas at 4 weeks (*n* = 8) are given; the amplitudes of a- and b-waves are indicated.

The functionality of the *Tulp1−/−* retinas treated at p2–3 with MD and HD of pAAV-*hRKP-Tulp1* was tested via ERG analysis at 4 and 6 weeks of age. In order to give injected retinas sufficient time to recover, ERG measurements are typically performed after 8–12 weeks post-injection. However, as the *Tulp1−/−* retina degenerates very rapidly, ERGs in this study were carried out at earlier timepoints of 4 and 6 weeks. Dark-adapted, rod-isolated (−25 dB), dark-adapted, mixed rod and cone (0 dB), and light-adapted, cone-isolated (0 dB) responses were detected; the ERG amplitudes measured in *Tulp1−*/*−* mice were much lower compared to wt mice. As the *Tulp1−/−* mice had very low ERG amplitudes and the dark-adapted, mixed rod and cone responses (Figure 4) were higher in amplitude compared to the rod- and cone-isolated responses (Supplementary Figure S2), we focused on this ERG measurement in our analysis (Figure 4). Both a-waves, which represent photoreceptor function and b-waves, which are mediated by bipolar cells were measured in pAAV-*hRKP-Tulp1* (without tracer AAV-*RhoP-EGFP*) treated *Tulp1−/−* eyes with MD and HD; uninjected wt and *Tulp1−/−* eyes were used as controls (Figure 4; *n* = 4–16). Dark-adapted, mixed rod and cone a- and b-wave amplitudes were 24.3 ± 13.5 μV and 52.2 ± 31.7 μV, respectively, in pAAV-*hRKP-Tulp1*-treated *Tulp1−/−* eyes with MD at 4 weeks (*n* = 8; Figure 4). While these were less than 15% of the wt levels (197.1 ± 63.1 μV and 378.7 ± 119.2 μV, respectively; *n* = 6), they were significantly different (*p* < 0.001; *t*-test) from the values in untreated *Tulp1−/−* eyes at 4 weeks (7.1 ± 7.0 μV and 9.4 ± 15.1 μV, respectively; *n* = 16). However, by 6 weeks, there were minimal dark-adapted, mixed rod, and cone responses detected in the MD-treated *Tulp1−/−* eyes (Figure 4). Additionally, there were minimal dark-adapted, mixed rod, and cone ERG responses detected with HD treatment at either 4 or 6 weeks. Amplitudes of dark-adapted rod- and light-adapted cone-isolated responses were minimal, often indistinguishable from the background in *Tulp1−/−* mice; as such, we did not analyze statistical significance between treated and untreated *Tulp1−/−* mice in these ERGs (Supplementary Figure S1).

## Discussion

We studied the effects of photoreceptor-targeted AAV-delivered supplementation of *Tulp1* in *Tulp1−/−* mouse retinas (Figure 2). While significant levels of *Tulp1* and TULP1 were achieved, there was no improvement in retinal histology (Figure 3). In contrast, minimal functional ERG benefit was determined at 4 but not 6 weeks (Figure 4). Overall, in spite of molecular restoration of *Tulp1* in a significant proportion of photoreceptor cells, only minimal and transient benefit was found in treated *Tulp1−/−* retinas and retinal degeneration was not halted.

AAV-delivered replacement/supplementation genes have been shown to provide benefit in many relevant IRD animal models (Ziccardi et al., 2019). In our own experience, in a similar scenario to the current *Tulp1* supplementation, *Rho−/−* mice (Humphries et al., 1997) with a photoreceptor specific gene (*Rho*) knocked-out were treated with AAV-delivered *RHO* replacement (Palfi et al., 2010, 2015). Significant and long-lasting structural and functional improvements were achieved using AAV2/5 (Palfi et al., 2010), 2/8 and 2/10 delivery (Palfi et al., 2015). Indeed, safety and efficacy of AAV-delivered gene therapies in animal studies have aided the progression of a number of such therapies into clinical trials (ClinicalTrials.gov); Luxturna for biallelic *RPE65* IRDs (FDA/EMA approvals in 2017–2018), *RPGR* and *PDE6B* supplementation for corresponding forms of RP, *RS1* supplementation for retinoschisis or *REP1* supplementation for choroideremia (ClinicalTrials.gov).

In light of these successes, supplementation of *Tulp1* in *Tulp1−/−* mouse retinas was considered to be a rational and feasible strategy. Indeed, in our study, *Tulp1* mRNA and protein expression in the AAV-*GRK1P-Tulp1*-treated *Tulp1−/−* retinas indicated a substantial restoration of *Tulp1*/TULP1, which was comparable to wt levels. The AAV supplemented TULP1 was localized not only in the cell bodies but in the segments and synaptic terminals of the photoreceptor cells at p20, which is also where the endogenous protein is localized in wt photoreceptors (Hagstrom et al., 1998; Ikeda et al., 2000). No obvious signs of major toxicity relating to the AAV delivery were observed in the treated *Tulp1−/−* mouse retinas, though the development of the ONL was possibly affected as detected by decreased thickness of ONL in treated wt retinas. The utilized AAV-*GRK1P-Tulp1* construct is based on previous designs and contains elements used before (Palfi et al., 2010; Chadderton et al., 2013). In particular, the utilized *GRK1* promoter is well tolerated in the mouse retina and provides photoreceptor specific expression (Beltran et al., 2010; McDougald et al., 2019). Yet, in spite of the molecular restoration of *Tulp1*/TULP1 levels in photoreceptors, we found minimal histological or functional rescue when comparing treated and control *Tulp1−/−* retinas. Photoreceptor degeneration was not halted as measured by ONL thickness but there was a small improvement in the ERG responses (both a- and b-waves) of the treated eyes at 4 weeks of age. However, by 6 weeks, there was no ERG response in the treated eyes. Notably, several studies have been carried out in *Tulp1−/−* mice since its creation (Hagstrom et al., 1998; Ikeda et al., 2000), yet there are no studies exploring *Tulp1* supplementation in these mice; suggesting that it may possibly be challenging to achieve therapeutic benefit. The supplementation strategy adopted here (e.g., expression construct, AAV serotype, expression levels as detailed above) was founded on prior knowledge and construct designs that provided benefit in other animal models, and therefore the overall therapeutic approach is unlikely to be the reason for inefficient rescue. As such, what factors could have contributed to the difficulties of rescuing the *Tulp1−/−* mouse retina with *Tulp1* supplementation?

The replacement AAV was delivered at p2–3, and it most likely takes a few days for the transgene expression to start up. In this regard, the earliest confirmation of expression of AAV2/5-supplemented transgene is 7 days post-delivery (Ezra-Elia et al., 2018). It is possible that *Tulp1* expression following the p2–3 delivery is too late and earlier presence of TULP1 is required for proper development and/or function of photoreceptor cells. In support of this hypothesis, it is important to note that expression of *Tulp1* has been detected in retinal progenitor cells (Milam et al., 2000) and embryonic retinas (Palfi et al., 2020).

While the exogenous TULP1 seemed to be present in the right compartments in photoreceptor cells at p20, note, that labeling in the cell bodies (Figure 3) was stronger than in wt mice and the differential distribution to the inner segments and synaptic terminals was not as distinct as in wt mice (Hagstrom et al., 1998; Ikeda et al., 2000; Palfi et al., 2020). For example, TULP1 is colocalized with f-actin (Xi et al., 2005) and MAP1B (Grossman et al., 2014) in the inner segments and the connecting cilium and is involved in trafficking of proteins such as rod- and cone opsins between the inner and outer segments (Grossman et al., 2011; Hagstrom et al., 2012). As these proteins are needed in large quantities in the outer segments, inadequate levels of TULP1 in the *Tulp1*-supplemented *Tulp1−/−* photoreceptors could have led to suboptimal levels of these proteins, which triggered degeneration, such as observed in *Rho−/−* retinas (Humphries et al., 1997). Similarly, TULP1 appears to be critical in synaptic transmission (Grossman et al., 2009). TULP1 quantities delivered to the synaptic terminals may not have been adequate for normal function and may have promoted degeneration in the treated *Tulp1−/−* photoreceptors.

Additionally, there is accumulating evidence indicating that *Tulp1* is not only expressed in photoreceptors but in other retinal cell types, such as cells in the INL, ganglion and RPE cells (Milam et al., 2000; Caberoy et al., 2010; Whitmore et al., 2014; Palfi et al., 2020). It is therefore possible that *Tulp1* may be important for the development and function of other retinal cell types; one hypothesis is that absence of *Tulp1* may affect these cells, which consequently may affect the development and function of photoreceptor cells and the retina. Notably, our expression system combining the AAV2/5 serotype and *GRK1P* provides *Tulp1* expression specifically in photoreceptors and not in other retinal cells. In this regard, it is interesting to mention that supplementation of *Cln3* in the inner retina but not photoreceptors resulted in therapeutic benefit in *Cln3*Δ*ex7/8* mice; a Batten disease model with a retinal phenotype and photoreceptors loss in patients (kleine Holthaus et al., 2020). Remarkably, in *rd1* mice with a mutation in the *Pde6b* gene (Bowes et al., 1990), a second mutation in *Gpr179* gene has been identified (Nishiguchi et al., 2015), which prevented full rescue with AAV-delivered *Pde6b* supplementation. Such a second mutation, while unlikely, could in theory also underlie the phenotype in the *Tulp1−/−* mouse retina and be responsible for inefficient rescue with *Tulp1* supplementation alone.

Many preclinical studies focused on development of gene-based medicines for IRDs have now progressed into clinical trials (ClinicalTrials.gov), with many other studies in the pipeline. With the ever-increasing number of IRD genes identified, the depth of understanding of the molecular pathogenesis of various IRDs (in patients and/or model systems) differs significantly. For example, *TULP1*-linked IRDs were originally identified as photoreceptor-based retinopathies, yet recent studies suggest both photoreceptor and non-photoreceptor expression of *Tulp1/TULP1*. Our current results in *Tulp1−/−* mice indicate that photoreceptor targeted *Tulp1* supplementation therapy may not be sufficient to provide significant and long-term benefit. As such, further studies are required to characterize this form of IRD in greater depth and to fine tune the gene augmentation strategy (for example providing earlier expression of *Tulp1* or targeting both photoreceptor and non-photoreceptor cells), which, in principle, should rescue the *Tulp1−/−* disease phenotype.

## Data Availability Statement

All datasets presented in this study are included in the article/Supplementary Material.

## Ethics Statement

The animal study was reviewed and approved by the Animal Research Ethics Committee, University of Dublin, Trinity College Dublin.

## Author Contributions

AP: concept, experimental design, experiments, figures and artwork, writing the manuscript, and grant support. AY: experimental design and experiments. SM-W: ERG, experimental design, and editing the manuscript. CS: pAAV-*CAGP-EGFP* plasmid and editing the manuscript. PH: concept and grant support. PK: ERG, AAV delivery, and experimental design. NC: AAV production, experimental design, and editing the manuscript. GF: concept, experimental design, editing the manuscript, and grant support. All authors read and approved the final manuscript.

## Conflict of Interest

The authors declare that the research was conducted in the absence of any commercial or financial relationships that could be construed as a potential conflict of interest.
