# Supplementary material for: AAV-Delivered Tulp1 Supplementation Therapy Targeting Photoreceptors Provides Minimal Benefit in Tulp1−/− Retinas
Source: Front Neurosci. 2020 Aug 27;14:891. doi: 10.3389/fnins.2020.00891 (PMC7482550; doi:10.3389/fnins.2020.00891)
Supplement: Supplementary file 1 [file Image_1.pdf]

Supplementary Material

Suppl. Figure 1.

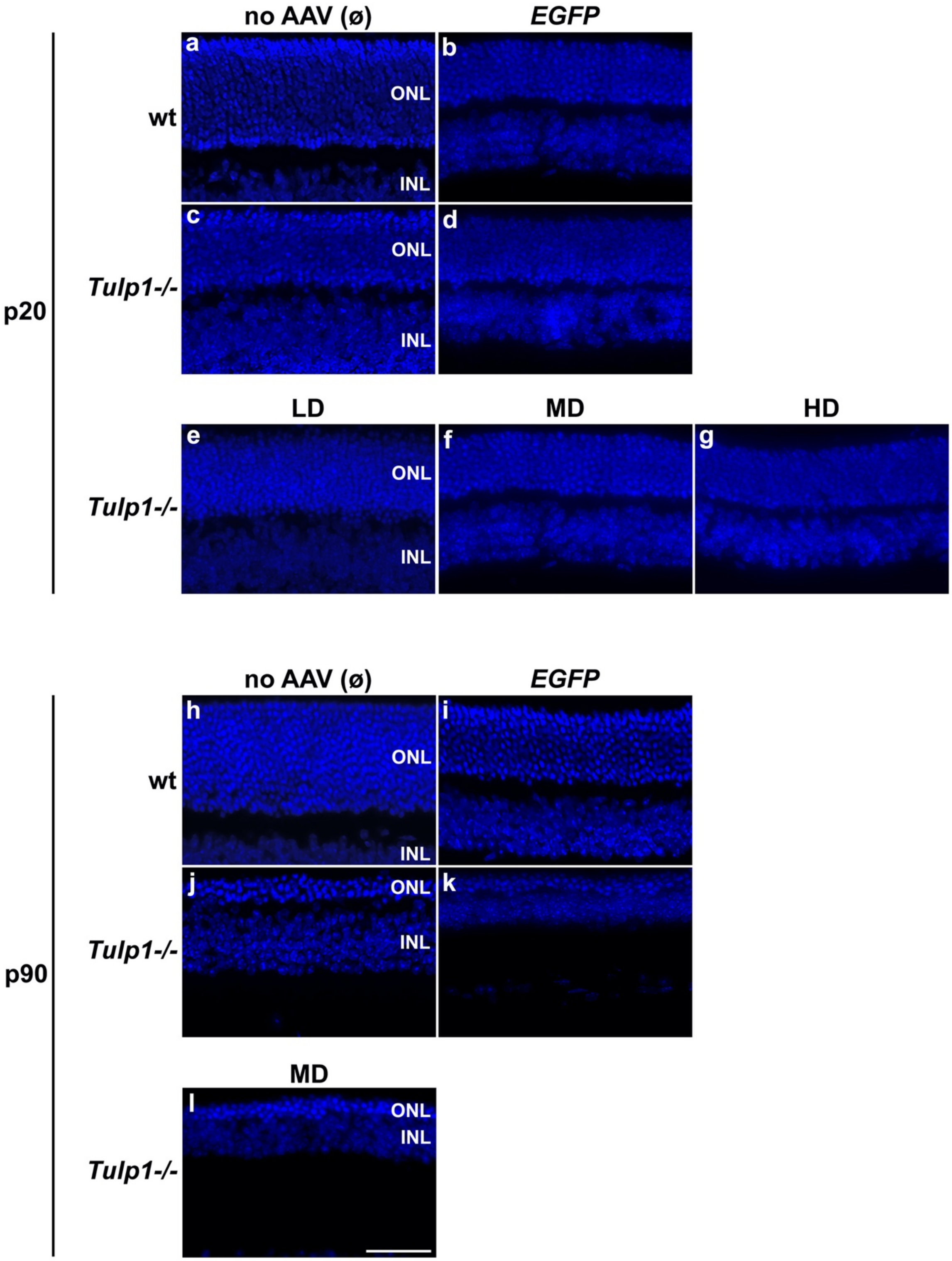

Suppl. Figure 1. Histological evaluation of *Tulp1* supplementation in *Tulp1-/-* retinas

Tulp1-/- pups were injected subretinally with LD, MD and HD doses of AAV-*GRK1P-Tulp1* + AAV-*RhoP-EGFP* as a marker (LD, MD, HD) or AAV-*RhoP-EGFP* (*EGFP*) as injection controls and their retinas analysed at p20 and p90 (n=4-6). Age-matched, uninjected (ø) wt and *Tulp1-/-* retinas were used as uninjected controls. Eyes were cryosectioned and nuclei counterstained with DAPI (blue). **a-b**: wt retinas analysed at p20, **c-g**: *Tulp1-/-* retinas analysed at p20, **h-i**: wt retinas analysed at p90, **j-l**: *Tulp1-/-* retinas analysed at p90. Scale bar (l): 40 µm. ONL: outer nuclear layer, INL: inner nuclear layer.

Suppl. Figure 2.

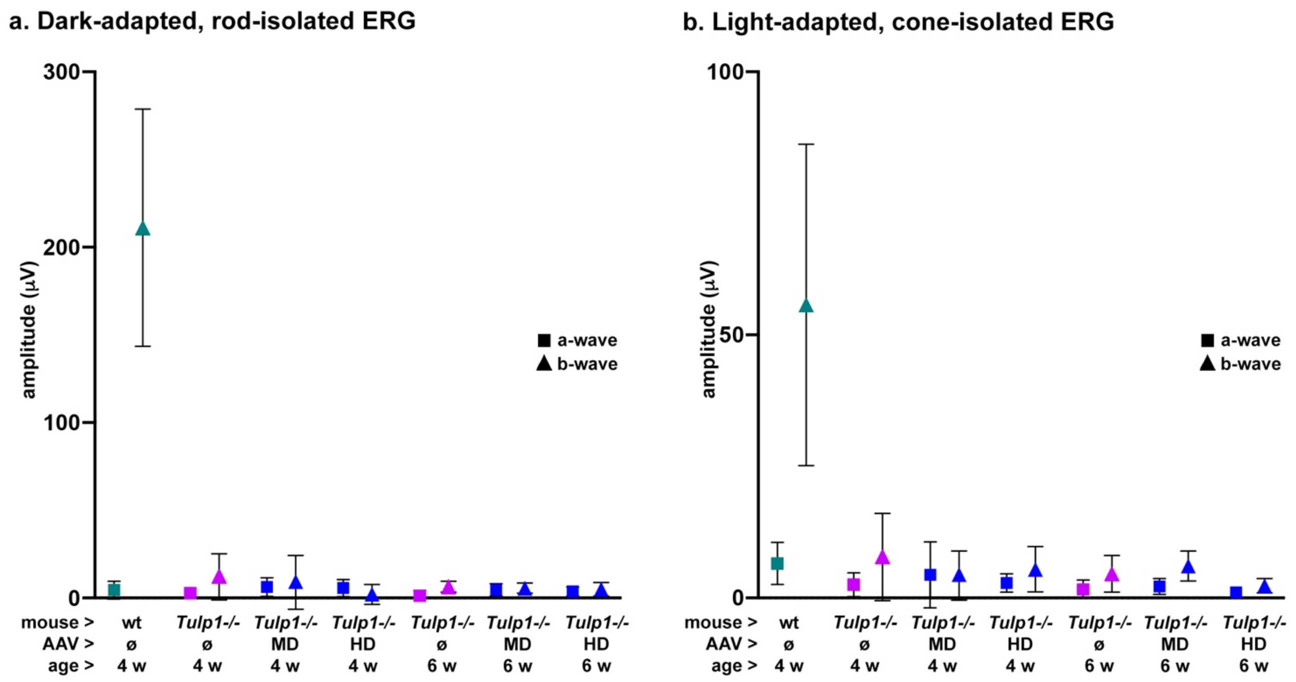

Suppl. Figure 2. Analysis of dark-adapted, rod-isolated and light adapted cone-isolated ERGs of *Tulp1* supplementation in *Tulp1-/-* retinas

Tulp1-/- mice were subretinally injected with MD or HD of AAV-*GRK1P-Tulp1* at p2-3; uninjected (ø) wt and Tulp1-/- retinas were used as controls (n=4-16); ERG analysis was performed at 4 and 6 weeks of age. **a**: Amplitudes of dark-adapted, rod-isolated (-25 dB) and **b**: light-adapted cone-isolated (0 dB) ERG responses are presented. a- (■) and b- (▲) waves are given for wt (green), untreated (magenta) and treated (blue) Tulp1-/- mice in dot plots; symbols represent mean±SD.
